# Therapeutic Effect of Chitooligosaccharide Tablets on Lipids in High-Fat Diets Induced Hyperlipidemic Rats

**DOI:** 10.3390/molecules24030514

**Published:** 2019-01-31

**Authors:** Di Yang, Canji Hu, Xiaoyi Deng, Yan Bai, Hua Cao, Jiao Guo, Zhengquan Su

**Affiliations:** 1 Guangdong Engineering Research Center of Natural Products and New Drugs, Guangdong Provincial University Engineering Technology Research Center of Natural Products and Drugs, Guangdong Pharmaceutical University, Guangzhou 510006, China; yangdi552@hotmail.com (D.Y.); hcj7319@163.com (C.H.); dxy1500718043@163.com (X.D.); angell_bai@163.com (Y.B.); 2 Guangdong Metabolic Diseases Research Center of Integrated Chinese and Western Medicine, Guangdong Pharmaceutical University, Guangzhou 510006, China; 3 School of Chemistry and Chemical Engineering, Guangdong Pharmaceutical University, Zhongshan 528458, China; caohua@gdpu.edu.cn

**Keywords:** antihyperlipidemic, chitooligosaccharide, gene difference expression, HMGCR, lipid metabolism

## Abstract

Chitooligosaccharide is beneficial for inhibiting dyslipidemia and reducing atherosclerotic and hyperlipidemic risk. The purpose of this study was to investigate the cholesterol-regulating effects and potential mechanisms of Chitooligosaccharide tablets (CFTs) in high-fat diet-induced hyperlipidemic rats. The results revealed that CFTs can regulate serum lipid levels in hyperlipidemic rats in a dosage-dependent manner. Synchronously, gene expressions related to cholesterol excretion were upregulated in a dosage-dependent manner, including cholesterol 7α-hydroxylase (CYP7A1), liver X receptor α (LXRA), peroxisome proliferation-activated receptor-α (PPARα) and low-density lipoprotein receptor (LDLR), whereas cholesterol synthetic gene expressions including 3-hydroxy-3-methylglutaryl-coenzyme A reductase (HMGCR) and sterol-responsive element binding protein-2 (SREBP2) were reduced. This work highlights that CFTs have potential as natural products to prevent and treat metabolic hyperlipidemia syndrome, probably due to the reduction of cholesterol biosynthesis and through cholesterol elimination; they also improve the pathological changes of liver tissue in rats, alleviate liver damage, maintain normal lipid metabolism in the liver, ameliorate hepatic glycolipid disorders and accelerate TC operation, and reduce blood lipid levels.

## 1. Introduction

With rapid economic development, diets have changed greatly in recent years, and fatty foods make up a large part of common daily diets. As a result, sustained high-fat diets lead to internal disorders of lipid metabolism in the body [1], which mainly manifest as low-density lipoprotein cholesterol (LDL) hypercholesterolemia, hypertriglyceridemia and low high-density lipoprotein cholesterol (HDL) [2]. Glycolipid metabolism disorders are associated with glucose and lipid metabolism disorders, inducing obesity, type-2 diabetes (T2DM), hypertension, dyslipidemia, nonalcoholic fatty liver, and atherosclerosis. Additionally, Lipoprotein levels are also disturbed or complicated with lipid metabolism disorders, accompanied by a reduction in HDL levels and an increase in total cholesterol (TC), triglyceride (TG) and LDL levels. Finally, obesity induced by excessive fat accumulation leads to hyperlipidemia. Hyperlipidemia is classified as one of the major risk factors leading to cardiovascular disease [3]. Although anti-hyperlipidemia drugs have a rapid lipid-lowering effect and excellent efficacy, their use has been limited due to their side effects [4]. Therefore, it is particularly important to find new drugs or functional foods that have lipid-lowering effects.

Chitooligosaccharide (COS) is an easily deacetylated and hydrolyzed product of chitosan, consisting of 2 to 10 glucosamine residues, 2-amino-2-deoxy-β-d-glucose, linked by β-1,4-glycosidic linkages [5]. Due to its biocompatibility, biodegradability and low toxicity, COS has drawn much attention in medical and pharmaceutical applications. Our previous studies showed that COS with a specific molecular weight improved lipid levels and total bile acid (TBA) levels in rats [6], and inhibited 3T3-L1 preadipocyte differentiation and enhanced hepatoprotective activities [7]. However, due to the small relative molecular weight of COS, it has a high solubility, which leads to extremely easy moisture absorption, making chitosan oligosaccharides physicochemically unstable. To improve the physical properties of its powder and to allow it to easily absorb moisture, COS film coating tablets (CFTs) were prepared, which consisted of 87.5% COS, 4% PPVP, 4% CaHPO4, 4% MCC, 0.5% magnesium stearate, 5% PVP solution (95% ethanol), 3% coating weight. In addition, the coating was evaluated by drug release characteristics and the effect was found to increase by 4% [8]. Therefore, CFTs with a specific molecular weight have shown improved stability and the ability to easily identified molecules [8]. However, the mechanisms of ameliorating lipid-metabolism disorders and the hepatic protection of CFTs remain unclear.

Many natural products have lipids-modifying effects mainly through regulating the cholesterol metabolism biosynthesis and cholesterol efflux. Cholesterol 7α-hydroxylase (CYP7A1), a rate-limiting enzyme of bile acid biosynthesis [9], played crucial regulatory roles in circulating TC and LDL-C levels. Sterol regulatory element-binding protein 2 (SREBP2) is a main regulator to control cholesterol biosynthesis gene expression [10]. 3-hydroxy-3-methylglutaryl coenzyme A reductase (HMGCR) controls the cholesterol elimination related genes expression, including low-density lipoprotein receptor (LDLR), peroxisome proliferator-activated receptor α (PPARα), liver X receptor alpha (LXRA). Regarding natural products, such as grape extract [11], barley sprout extract [12] and Lonicera caerulea berry extract [10] could accelerate the conversion of cholesterol to bile acids and increase the excretion of bile acid in feces by regulating CYP7A1, SREBP2 and HMGCR gene expression, respectively.

Therefore, the aim of the present work was to investigate the effects of CFTs, which expressed improved stability and easily identified molecules, prepared according to our previous study [13], on lipoprotein levels and cholesterol concentrations. According to the differential gene expression of liver tissues in hyperlipidemic rats, we investigated the antihyperlipidemic effects of CFTs, and these findings illustrated that CFTs can ameliorate the disorders of lipid metabolism, suggesting that CFTs could serve as an alternative functional food for the prevention and treatment of hyperlipidemia.

## 2. Results

### 2.1. Weight Gain, Food Intake, Fat Ratio, and Liver Index

**Figure 1:**
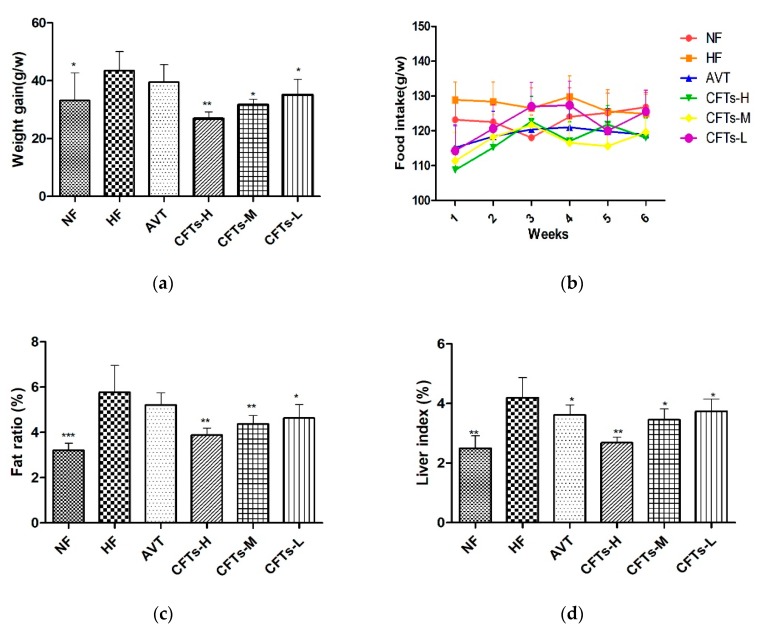
The main index of rats. (**a**) weight gain; (**b**) food intake; (**c**) fat ratio; (**d**) liver index. The data are presented as the means ± SD (*n* = 10). Compared to HF group, * *p* < 0.05; ** *p* < 0.01; *** *p* < 0.001.

To understand the possible effects of lipids modification, we determined six-week body weight gains and food intake. CFTs treatment significantly decreased weight gain rates compared to HF groups (Figure 1a). CFTs treatment at 1200 mg/kg·d in high-fat induced diets significantly decreased body weight gains (*p* < 0.01). Similarly, CFTs treatment at 600 and 300 mg/kg·d decreased body weight gains (*p* < 0.05). As shown in Figure 1b, there were no significant differences in all groups. These results indicate that CFTs decreased weight gain rates without influencing appetite in high-fat diet-induced rats. However, the treatment with AVT showed no significance in body weight gains. These results suggest that CFTs reduced weight gain rates in a dose-dependent manner.

As shown in Figure 1c, rats in the HF group with high-fat diets had a higher fat ratio than those in the NF group (*p* < 0.001); rats in the former group also showed a high percentage of white adipose tissue in HF groups. In addition, the treatments with CFTs at 1200, 600 and 300 mg/kg·d significantly decreased the body ration (*p* < 0.01, *p* < 0.01 and *p* < 0.05, respectively) compared with the HF group, while there was no significance in the AVT group. Liver indexes are shown in Figure 1d. Notably, our results showed that rats in the NF group with normal food diets exhibited a significant lowering effect on liver index compared to those of the HF group with high-fat diets (*p* < 0.05). The treatment experiments with CFTs and the AVT group markedly reduced the liver index compared to HF group, and CFTs treatment groups exhibited a dose-dependent effect on the liver indexes (CFTs-H: *p* < 0.01, AVT: *p* < 0.05). These results suggest that CFTs inhibit the accumulation of fat pad and reduce the fat body ratio in a dose-dependent manner; finally, the CFTs treatment groups showed slightly better results than the AVT group in reducing the fat body ratio.

Obesity often leads to diseases such as abnormal lipid metabolism and hyperlipidemia [14,15]. These results demonstrate that CFTs efficiently reduced the weight gain and indirectly reduced the risk of hyperlipidemia in rats given high fat diets by inhibiting the accumulation of fat pad in high-fat diet-induced rats.

### 2.2. Serum and Liver Lipid Levels in Rats

**Figure 2:**
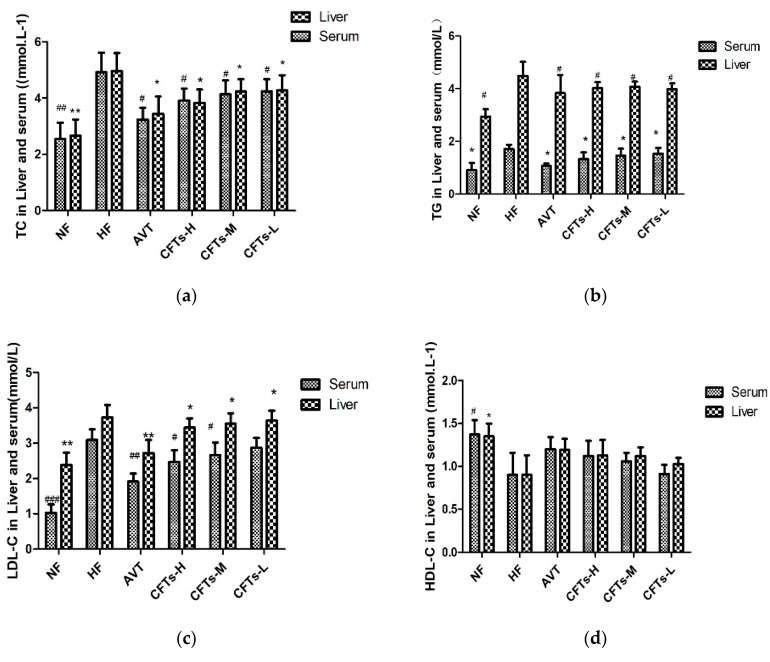
The effect of CFTs on lipid levels in the serum and liver. (**a**) TC levels in serum and liver; (**b**) TG levels in the serum and liver; (**c**) LDL-C levels in the serum and liver; (**d**) serum and liver HDL-C levels. These data are presented as the means ± SD (*n* = 10). (*^,#^) Significant difference at *p* < 0.05, (**^,##^) significance difference at *p* < 0.01 and (^###^) significance difference at *p* < 0.001 VS the HF group.

Studies have shown that the high-fat diets lead to increases in TC, TG, LDL levels and a reduction in HDL levels [16]. As shown in Figure 2a–d and Table 1, serum and liver TC, TG, LDL levels of the NF group were significantly lower than those of the HF group (serum LDL: *p* < 0.001, serum and liver TC and TG: *p* < 0.01), showing that a hyperlipidemia rats model was established successfully. Compared with the HF group, treatments with the CFTs groups showed that serum levels significantly decreased, and serum levels in CFTs-H, CFTs-M, and CFTs-L treatment groups were significantly lower than those in the HF group, with TCs being reduced by 20.53%, 15.85%, and 13.82%, respectively, TG decreasing by 37.28%, 13.02%, and 9.47%, respectively, and LDL-C decreasing by 23.10%, 17.41%, and 11.39% (*p* < 0.05), respectively. However, there were no significant increases in HDL level in the CFTs treatment groups. These results suggest that CFTs can improve the serum lipid levels in a dose-dependent manner.

**Figure 3:**
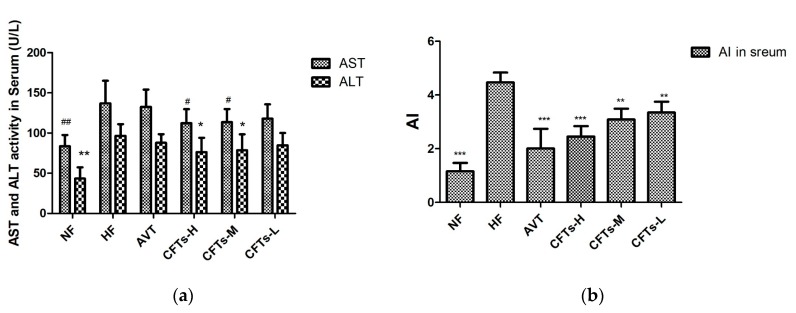
Serum aminotransferase and arteriosclerosis levels. (**a**) AST and ALT index in serum; (**b**) AI index in serum. The data are presented as the means ± SD (*n* = 10). (*^,#^) Significant difference at *p* < 0.05, (**^,##^) significance difference at *p* < 0.01 and (^###^) significance difference at *p* < 0.001 VS the HF group.

Atherosclerosis often leads to thrombosis and blood supply disorders. Atherogenic Index (AI, AI = (TC − HDL)/HDL) was considered as a marker of cardiovascular disease [17]. As shown in Figure 3 and Table 1, the serum AI level in the CFTs-H, CFTs-M, and CFTs-L treatment groups were significantly lower than those of the HF group, with AI decreasing by 54.55% (*p* < 0.001), 47.07% (*p* < 0.001) and 25.47% (*p* < 0.01), respectively. These results imply that CFTs decrease serum AI values in a dose-dependent manner, and ameliorate serum HDL-C levels in high-fat diet-induced rats.

Liver damage and hepatotoxicity are the main symptoms of hyperlipidemia [18,19]. Additionally, the serum ALT and AST activities are thought to be the main toxicological parameters for liver function [20]. As shown in Figure 3, the serum AST and ALT activities of CFTs-H and CFTs-M group were significantly lower than those of the HF group (*p* < 0.05), proving that CFTs has no toxicity in the liver.

### 2.3. Serum MDA, SOD in Rats

**Figure 4:**
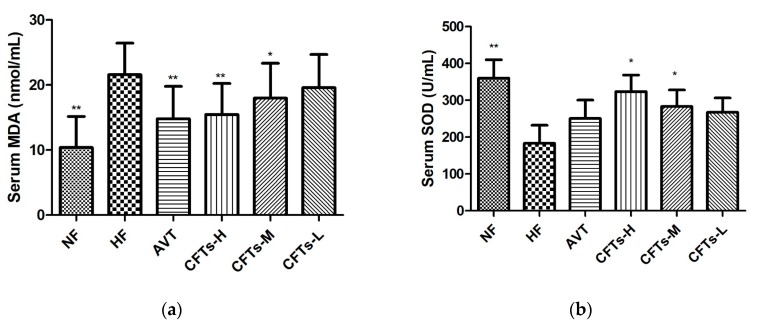
Serum MDA, SOD in rats. (**a**) Serum MDA; (**b**) Serum SOD. (*) Significant difference at *p* < 0.05 and (**) signficant difference at *p* < 0.01 VS the HF group. Malondialdehyde (MDA) and Superoxide dismutase (SOD) are important factors affecting the amount of lipid deposition in the liver.

Malondialdehyde (MDA) is the ultimate product of lipid oxidation and can indirectly measure the extent of damage to the biofilm system. In Figure 4a, it can be seen that the serum MDA content of the experimental rats in the high-fat groups is higher than that of NF. In the NF group, experiments have shown that high-fat diets increase the body’s oxidative stress response. In addition, the serum MDA value in the CFTs-H, CFTs-M group was significantly lower than that in the HF group, indicating that CFTs can effectively inhibit the level of oxidative stress in rats.

Superoxide dismutase (SOD) is widely distributed in various organisms to protect cells from damage by removing superoxide anions from living organisms. It can be seen from Figure 4b that the high-fat diet significantly reduced the SOD activity of the rat liver, compared with the NF group (*p* < 0.05), indicating that the high-fat diet reduced the liver’s antioxidant capacity, while also increasing the body’s oxidative stress response, easily causing the disorder of lipid metabolism in rats. The serum SOD of the rats in the NF group was smaller than that of the other groups, and the serum SOD in the CFTs-H and CFTs-M groups was significantly higher than that in the HF group, showing that the high-fat diet increased the oxidative stress response in rats, and that CFTs could increase the serum SOD activity.

### 2.4. TC, TG and TBA in Feces

**Figure 5:**
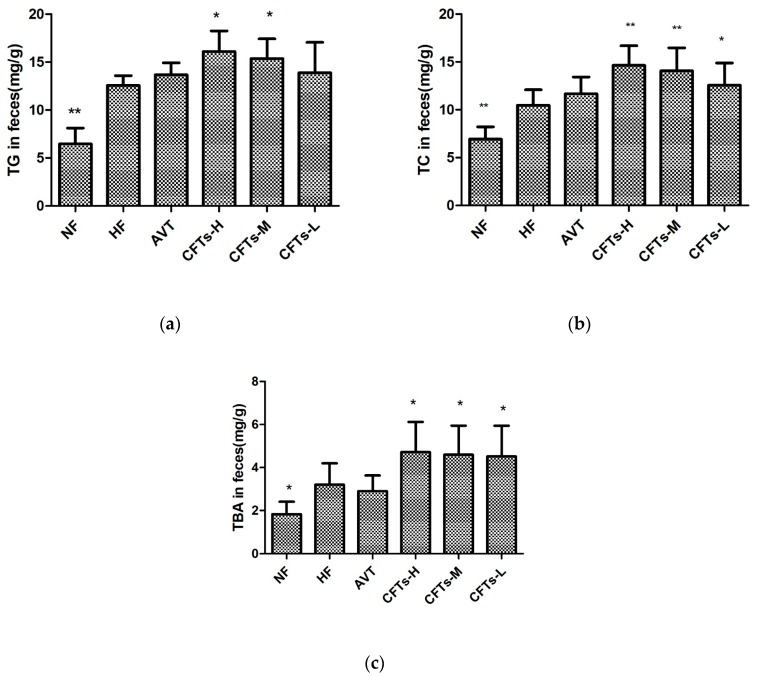
The main index in rats feces. Fecal TC (**a**), TG (**b**) and TBA (**c**). These data are presented as means ± SD (*n* = 10). Compared to HF, * *p* < 0.05; ** *p* < 0.01.

To determine the reduction of lipid absorption, we investigated TC, TG and TBA levels in rat feces. As shown in Figure 5 and Table 1, the fecal TC levels showed a significant increase in the CFTs group, especially rats of the CFTs-H and CFTs-M group (*p* < 0.01), compared with HF group. The fecal TG levels in the CFTs-H and CFTs-M group (*p* < 0.05) were significantly higher than those in the HF group, and the same as the fecal TBA levels in treatments with the CFTs group. To reduce fat absorption, excessive cholesterol was transported from peripheral tissue to the intestine, where it promoted excretion of bile acids. These results indicate that CFTs effectively ameliorates lipid metabolism by accelerating the excretion of cholesterol in the feces.

### 2.5. Liver and Kidney Histology

**Figure 6:**
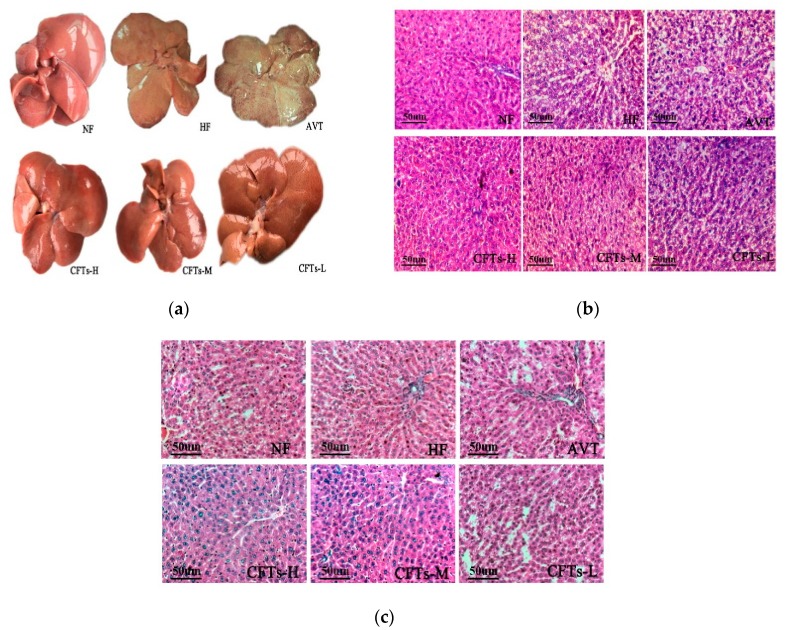
The whole liver (**a**), slices of liver (**b**) and kidney (**c**) from different groups of rats (200×) after 6 weeks of treatment. Tissue sections were stained with hematoxylin and eosin (H & E).

Figure 6a shows images of rats livers; the NF group was bright red in color, soft, sharply-defined and low in volume, whereas intumescent tissue, hypertrophic edges and white fat granules were obviously observed in the HF group, suggesting that a severe high fat diet promotes the process of fatty liver-like diseases in rats. Treatment with CFTs showed improvement in liver steatosis, with bright red and slight pale color, indicating the presence of fewer white fat particles. Figure 6b shows the result of a histological examination of liver; the NF group showed normal histological structures, whereas the HF group displayed severe lipid droplets and partly infiltrated the inflammatory cells, proving that a high-fat diet can induce liver steatosis in rats. Treatment in the CFTs groups significantly decreased lipid droplets in livers with different degrees, particularly in the CFTs-H group. Figure 6c shows the result of a histological examination of kidney; the CFTs groups showed complete glomerular structures. No pathological changes were found in the renal sac, mesangial cells and stroma, capillary basement membrane and supporting cell population, similar to that of the NF group, indicating that different doses of CFT were harmless and non-toxic to rat kidney. These results suggest that one of the mechanisms of CFTs hypolipidemic properties may be to improve the pathological changes of liver tissue in rats, alleviate liver damage, maintain normal lipid metabolism in the liver, accelerate liver glycolipid and TC operation, and reduce blood lipid levels.

### 2.6. Adipose Tissues Histology

**Figure 7:**
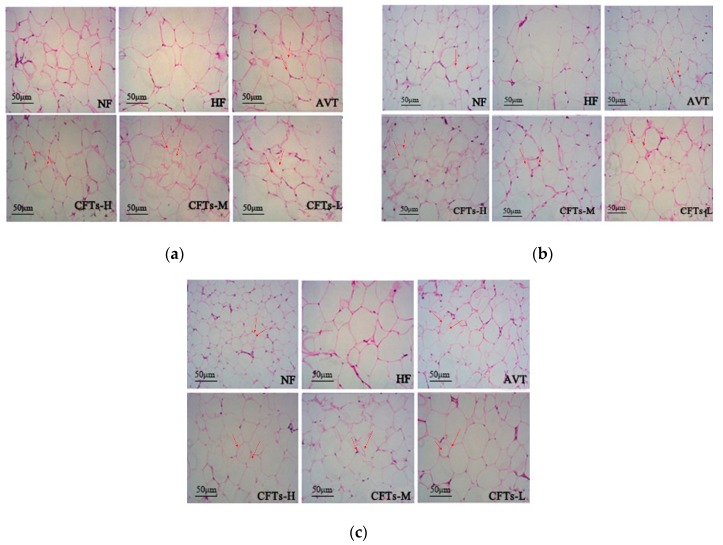
Adipose tissues histology. (**a**) perirenal adipose tissues, (**b**) subcutaneous adipose tissues, and (**c**) epididymal adipose tissues from different groups of rats (200×) after 6 weeks of treatment. Tissue sections were stained with hematoxylin and eosin (H & E).

Figure 7 shows a histological examination of perirenal adipose tissues, and subcutaneous and epididymal adipose tissues in rats, respectively. The perirenal and subcutaneous fat cells of HF were significantly more hypertrophic than those of the NF group, and the treatment with CFTs groups showed a different degree of reduction in the size of adipocytes, suggesting that CFTs have an anti-hyperlipemia activity by reducing the accumulation of white adipose tissues.

### 2.7. Differential Gene Expression Analysis in Liver

**Figure 8:**
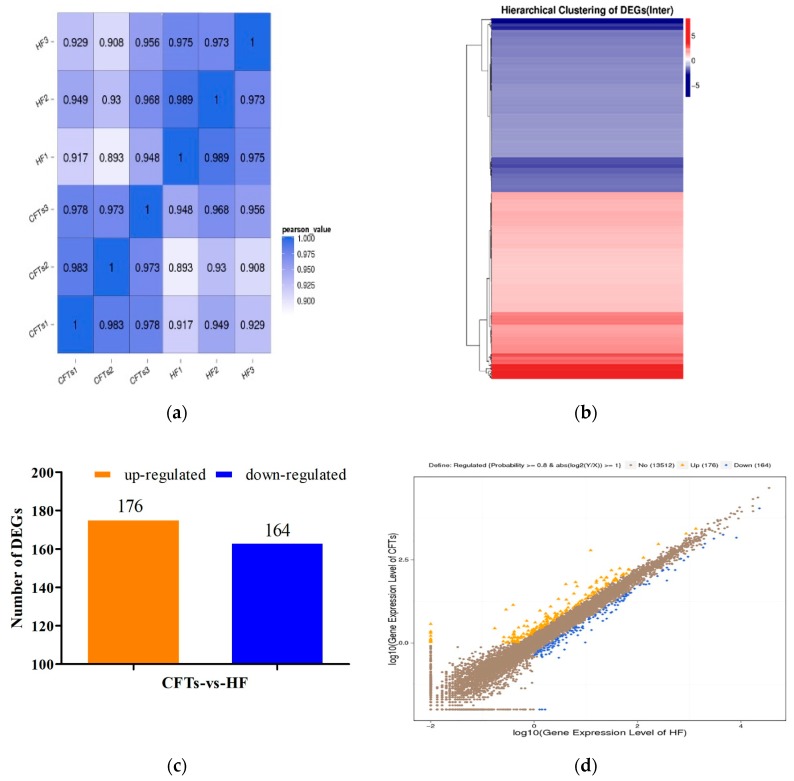
Differential gene expression analysis (HF vs CFTs). (**a**) Heatmap of correlation coefficient values; (**b**) Intersection heatmap of DEGs; (**c**) Statistic of differentially expressed genes; (**d**) Scatter plots of all expressed genes.

To understand the molecular mechanism of the anti-hyperlipidemic effects of CFTs, differential gene expression and GO enrichment analysis of liver in HF, CFTs group were conducted. As shown in Figure 8a–d, differential gene expressions were detected in the CFTs groups and the HF group. Figure 8a directly indicates good biological repeatability; Figure 8b–d shows that 176 and 164 genes were up-regulated and down-regulated, respectively. These results indicate that CFTs can regulate the expression of genes in hyperlipidemic rats.

**Figure 9:**
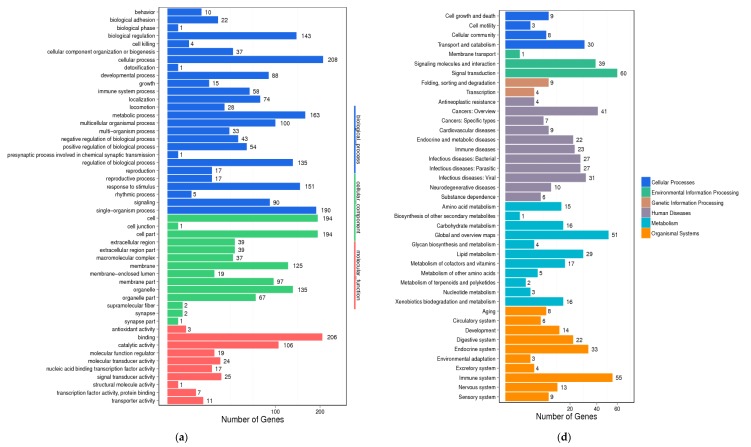
Differential gene expression analysis (HF vs CFTs). (**a**) GO functional classification on DEGs; (**b**) KEGG classification on DEGs.

Differential gene expression showed a large difference. The GO enrichment analysis is displayed in Figure 9, suggesting that differentially-expressed genes of rats in the CFTs-administered groups were related to biological regulation, cellular processes and molecular function. These results show that enriched genes were mainly related to biological procedures, including fatty acid metabolism, lipid metabolism and steroid metabolism.

### 2.8. CFTs Affects the Expressions of Lipid Metabolism Target Genes in Liver

**Figure 10:**
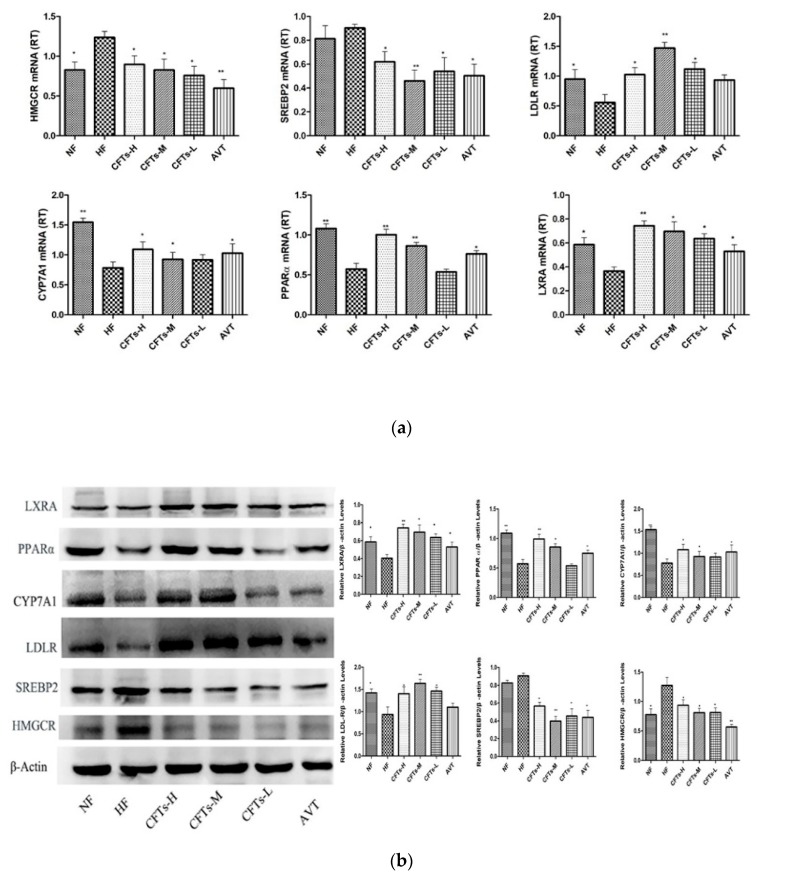
Effects of CFTs on hepatic genes expression. (**a**) Hepatic genes expression fold change in rats treated with CFTs for 6 weeks; (**b**) CFTs regulated expression levels of lipogenic genes. Compared to HF, * *p* < 0.05, ** *p* < 0.01.

Relative folds in the levels of HMGCR, SREBP2 and LDLR activity in the livers of rats were quantified using real-time RT-PCR after 6 weeks with therapeutic administration (Figure 10). As shown in Figure 10a, treatments with the CFTs group significantly decreased HMGCR mRNA expression (*p* < 0.05) compared to that of HF group. Similarly, SREBP2 mRNA expression was significantly reduced (*p* < 0.05 or *p* < 0.01). However, LDLR mRNA expression was increased by CFTs. The protein expression profiles of HMGCR, SREBP2 and LDRL in the livers were the same as those of the mRNA profiles (Figure 10a). The AVT group showed similar effects on the regulatory gene and protein expressions, including HMGCR, SREBP2 and LDLR expression. These results indicate CFTs can inhibit hepatic cholesterol synthesis by down-regulating HMGCR and SREBP2 expression and upregulating the LDLR expression.

Based on the differential expression of lipid metabolism, several key differential genes were confirmed for the molecular function, including CYP7A1, LXRA and PPARα expression. As shown in Figure 10a, CYP7A1, LXRA and PPARα mRNA expression in the liver of CFTs-H groups was up-regulated compared to HF group (*p* ≤ 0.01), and the mRNA expression levels of the CFTs-L and CFTs-M groups were significantly up-regulated. The AVT group showed similar effects. The protein expression profiles of CYP7A1, LXRA and PPARα protein expression in the liver were the same as those of the mRNA profiles (Figure 10b), suggesting that CFTs promote hepatic cholesterol excretion by upregulating CYP7A1, LXRA and PPARα expression.

## 3. Discussion

COS is a promising anti-obesity and hypocholesterolemic agent in rats. As our previous studies stated, it indirectly reduced the risk of hyperlipidemia [21]. In the present work, we showed that CFTs administration effectively alleviated the hyperlipidemic symptoms of high-fat diet-induced rats, including the improvement of body weight gain, liver index, size of adipose tissue, serum lipids levels. Compared with the HF group, the administration of 6-week CFTs led to marked reductions in serum TC, TG and LDL levels, i.e., the same as the effects of serum AST and ALT levels in high-fat diet-induced rats, especially in the CFTs-H group (*p* < 0.05), implicating that CFT has no damaging and toxic effects on liver function, and minimizes cholesterol residue caused by high-fat diets. Also, the results indicated that CFTs can ameliorate the fatty degeneration degree of the liver, and modulate the expression levels of lipid metabolism-related genes in a dose-dependent manner. We demonstrated that CFTs improved lipid-lowering activity by upregulating CYP7A1, LXRA and PPARα gene expression to facilitate cholesterol exclusion via conversion into bile acid, and downregulating HMGCR, SREBP2 and LDLR genes expression to reduce the cholesterol biosynthesis.

To investigate the molecular mechanism of the anti-hyperlipidemic effect of CFTs, differential gene expression and GO enrichment analyses were conducted. From the analyzed result, 340 genes were enriched, expression modifications of 176 genes were observed, and 164 genes were downregulated. These enriched genes were mainly related to biological procedures, including bile acid metabolism, inflammation development, steroid metabolism and fatty acids metabolism. According to gene differential expressions in fatty acid and lipid metabolism, we found some interesting genes, including that CYP7A1, HMGCR, LXRA, SREBP2, LDLR gene expressions in liver might be the target of CFTs lipid-lowering process.

Cholesterol balance was reflected in cholesterol absorption, synthesis, transport, and excretion [22]. Cholesterol biosynthesis was mainly regulated by HMGCR, the rate-limiting enzyme, then cholesterol was metabolized to bile acid by CYP7A1 to reduce cholesterol accumulation [11,23,24]. Our ttudy showed that LXRA mediated the transcription of CYP7A1 and cholesterol excretion in the form of bile acids in rats [25], which could activate LDLR expression. PPARα is considered as a nutritional sensor that orchestrates lipoprotein metabolism and hepatic lipogenesis in liver [26]. SREBP-2 was reported to primarily regulate cholesterol synthesis gene expression, including CYP7A1, HMGCR and cholesterol uptake via LDLR [27,28,29]. Thus, HMGCR inhibitors can be used to lower cholesterol levels, and the inhibition of SREBP2 and HMGCR activities could be targets for effectively inhibiting cholesterol biosynthesis and reducing cholesterol accumulation in vivo.

Above all, we have shown that CFTs ameliorate disorders of lipid metabolism by upregulating CYP7A1, LXRA and PPARα gene expression, which facilitated the cholesterol excretion in the form of bile acid. In addition, CFTs also downregulated HMGCR and SREBP2 gene expression and upregulated LDLR gene expression, which is an effective way to control serum cholesterol synthesis levels. Together, the results in the present work reveal that CFTs might be promising natural products for the prevention and treatment of hyperlipidemia.

## 4. Materials and Methods

### 4.1. Materials

Commercial samples of CFTs (molecular weight ≤ 1K Da; degree of deacetylation, 95.6%, lot: 160927C) were purchased from Qingdao (Shandong, China). Atorvastatin Calcium tablets were bought from Pfizer Pharmaceutical Company Limited (Dalian, Liaoning, China). Povidone and carboxymethylcellulose sodium were purchased from Tianjin Kermel Chemical Reagent Co., Ltd. (Tianjin, China). TC, TG, HDL, LDL, Aspartate Aminotransferase (AST) and Alanine Aminotransferase (ALT) were measured using commercially-available kits (Biosino, Beijing, China). CFTs were prepared in the laboratory.

### 4.2. Animals and Diets

Male Sprague-Dawley rats (200 ± 20 g; 8 weeks; No.44007200034526) were purchased from the Guangdong Medical Laboratory Animal Center (GMLAC, Guangzhou, China). Animals were fed in a specific pathogen-free room that was maintained at 22–25 °C and 50–70% humidity under a day-night rhythm. Animal experimental protocols were approved by the Institutional Animal Care and the Use Committee of Guangdong Pharmaceutical University (Guangzhou, China).

Rats were fed to adapt to the new environment for 1 week before experiments. Then, the rats were randomly divided into two groups: normal control group (NF, *n* = 15), hyperlipidemic control group (*n* = 65), which were fed a 6-week regular diet and a high-fat diet (52.6% regular diet, 20.0% sucrose, 15.0% lard, 1.2% cholesterol, 0.2% bile salts, 10% casein, 0.6% calcium hydrophosphate, 0.4% mountain flour) respectively to obtain the hyperlipidemic model. Food and water were freely available to all rats.

Blood samples were taken from the orbital vein under ether anesthesia to determine serum TG, TC, and LDL-C levels during the modeling period, and the differences of TC, TG, or LDL-C levels were significant as models for the model group compared to NF group. Then, the hyperlipidemia rats were randomly divided into 5 groups (*n* = 10 rats per group): high-fat diet group (HF); high-fat diet administered with atorvastatin (7 mg/kg day) (AVT), which was clinically used to treat hypercholesterolemia extensively, and has been shown to be safe; high-fat diet administered with CFTs (300 mg/kg day) (CFTs-L); high-fat diet administered with CFTs (600 mg/kg day) (CFTs-M); and high-fat diet administered CFTs (1200 mg/kg day) (CFTs-H) according to our previous study [6]. All samples were dissolved in aqueous solution and orally administered by gavage at a dose of 1 mL/100 g·d for six weeks. During the diet intervention, body weight and food intake were measured each week. At the end of the administration, we measured the rats’ body weight, the fat pad and the liver weight, and calculated the liver index (liver index = liver weight/body weight) and fat ratio (fat ratio = fat pad weight/ body weight).

### 4.3. Serum Biochemical Analysis

Serum was obtained from blood by centrifugation (4 °C, 3500 r/min, 10 min), and stored at −80 °C. Serum TG, TC, LDL, and HDL levels were investigated with commercial assay kits and an automated biochemistry analyzer BC200 instrument (BC200, Beijing Precil Instrument Co. Ltd., Beijing, China). ALT and AST activities were analyzed using commercial kits.

### 4.4. Histological Staining

The liver, kidney, white epididymal, perirenal, and subcutaneous adipose tissues were taken and fixed in 12% formaldehyde solution, dehydrated with ethanol, embedded with paraffin and 3–5 μm thick sections for staining with hematoxylin and eosin (H & E), then observed under an optical microscope at 200× magnification.

### 4.5. Gene Expression Tag Profiling

The liver RNA in the HF and CFTs groups were extracted using Agilent RNA 6000 Pico kits (Agilent Technologies, Santa Clara, CA, USA). The concentration and quality were measured with Agilent 2100 instrument (Agilent Technologies). The Beijing Genomics Institution conducted the digital gene expression Tag Profiling. Differentially-expressed genes were screened with NOISeq method and analyzed with a Gene Ontology method for clustering genes.

### 4.6. Quantitative RT-PCR

Total RNA was isolated from rat liver tissues using TRIzol reagent (Invitrogen, Inc., Carlsbad, CA, USA). Single-stranded cDNA was synthesized with the PrimeScriptTM RT Reagent kit with gDNA Eraser (TaKaRa, Lot: D413KA5332, Shiga, Otsu, Japan). The cDNA products were amplified using real-time RT-PCR and the TaKaRa SYBR Premix Ex Taq™ kit (TaKaRa, Lot: AK7103, Shiga, Otsu, Japan). Primer sequences are listed in Table 2 and analysis software (Applied Biosystems, Carlsbad, CA, USA). Data were normalized to actin mRNA. The relative quantification of mRNA expression was analyzed using the ΔΔ*C*t method.

### 4.7. Western Blotting

Total proteins were extracted from the rats livers using cold RIPA Lysis Buffer (Beyotime, Lot: P0013B, Shanghai, China), with the addition of protease and phosphatase inhibitor cocktails (Beyotime, Lot: ST506, Shanghai, China) centrifugated twice at 12,000× *g* for 20 min 4 °C, protein normalization using the BCA Protein Assay Kit (Beyotime, Lot: 0907A16, Shanghai, China). Equal amounts of protein samples (40.0 µg) were mixed with buffer (5×, Beyotime, Lot: P0015, Shanghai, China), then the mixture was deformed at 99 °C for 10 mins and cooled. Protein samples were electrophoresed with sodium dodecyl sulfate-polyacrylamide gel electrophoresis (SDS-PAGE) using a 6–15% (*v*/*v*) gradient polyacrylamide gel, and the separated protein bands were transferred onto polyvinylidene difluoride membranes (Millipore Corp., Billerica, MA, USA). Sealed with 5% bovine serum albumin (BSA), the membranes were reacted with rat primary antibodies, including HMGCR, CYP7A1, SREBP2, LDLR, LXRA, and PPARα polyclonal antibodies (Proteintech, Inc., Wuhan, China), and rabbit anti-β-actin (Biosynthesis Biotechnology Co., Ltd., Beijing, China), followed incubation with Goat Anti-rabbit Immunoglobulin G/horseradish peroxidase (Goat Anti-rabbit IgG/HRP, Biosynthesis Biotechnology Co., Ltd., Beijing, China). The resulting blots were reacted with ECL Detection Reagent (Millipore Corp., Billerica, MA, USA) and detected with a chemiluminescence imaging system (Sage Creation, Beijing, China). The gray value of the quantified protein bands was used for the Lane 1D gel image software (Sage Creation, Beijing, China).

### 4.8. Statistical Analysis

All data were performed as the means ± SD. Differences among groups were determined using a one-way ANOVA test using SPSS software (SPSS Inc., Chicago, IL, USA), *p* < 0.05 was considered significant. Analyses were performed using Graph Pad Prism version 7.0 (GraphPad Software version 7.0, San Diego, CA, USA).

## 5. Conclusions

This work was the first to demonstrate that CFTs have latent functions as a natural product for the prevention and treatment of hyperlipidemia, probably due to the reduction of cholesterol biosynthesis and the addition of cholesterol elimination. The potential cholesterol regulation mechanisms of CFTs could ameliorate CYP7A1 activity to increase the cholesterol excretion in the form of bile acids and control the de novo synthesis of cholesterol by inhibiting HMGCR and SREBP2 activities, also improving the pathological changes of liver tissue in rats, alleviating liver damage, maintaining normal lipid metabolism in the liver, accelerating the liver glycolipid and TC operation, and reducing blood lipid levels. Although further molecular mechanisms are required for research in the future, these obtained findings about dietary interventions using natural agents like CFTs might be prove to be useful in research on hyperlipidemia.

## 6. Patents

Su, Z., G. J, and Q. Yang. Chitosan Oligosaccharide Tablet and Preparation Method Thereof. ZL201510988018.1 (2018).

## Figures and Tables

**Table 1:** The serum, liver and fetal lipid levels in high-fat diet rats.

Index	NF	HF	AVT	CFTs-H	CFTs-M	CFTs-L
Serum (mmol/L)						
TC (mmol/L)	2.44 ± 0.52 **	4.92 ± 0.63	3.24 ± 0.37 **	3.91 ± 0.39 *	4.14 ± 0.45 *	4.24 ± 0.40 *
TG(mmol/L)	0.95 ± 0.32 **	1.69 ± 0.15	1.06 ± 0.08 **	1.33 ± 0.25 **	1.47 ± 0.25 *	1.53 ± 0.20
HDL-C (mmol/L)	1.51 ± 0.31 *	0.82 ± 0.28	1.06 ± 0.32	1.06 ± 0.32	1.56 ± 0.29	0.96 ± 0.45
LDL-C (mmol/L)	1.11 ± 0.25 ***	3.16 ± 0.25	1.89 ± 0.22 *	2.43 ± 0.32 *	2.61 ± 0.35 *	2.80 ± 0.22
AI	0.96 ± 0.55 ***	5.26 ± 1.77	1.79 ± 0.44 ***	2.39 ± 0.18 ***	2.78 ± 0.43 **	3.92 ± 0.75 **
AST (U/L)	83.06 ± 13.86 **	138.22 ± 27.98	134.02 ± 21.13	114.20 ± 16.78 *	115.52 ± 15.65	118.46 ± 17.80
ALT (U/L)	44.88 ± 13.56 **	92.88 ± 11.35	85.52 ± 8.76	75.00 ± 17.62 *	78.64 ± 19.76	85.18 ± 15.23
Liver (mg/g)						
TC	2.66 ± 0.58 ^##^	4.97 ± 0.64	3.44 ± 0.62 ^#^	3.83 ± 0.48 ^#^	4.24 ± 0.44 ^#^	4.28 ± 0.53 ^#^
TG	2.94 ± 0.28 ^#^	4.48 ± 0.54	3.84 ± 0.68 ^#^	4.02 ± 0.23 ^#^	4.07 ± 0.2 ^#^	3.98 ± 0.22 ^#^
HDL-C	1.35 ± 0.15 ^#^	0.9 ± 0.23	1.19 ± 0.13	1.13 ± 0.18	1.12 ± 0.1	1.03 ± 0.07
LDL-C	2.38 ± 0.35 ^##^	3.73 ± 0.35	2.72 ± 0.37 ^##^	3.44 ± 0.26 ^#^	3.55 ± 0.3 ^#^	3.64 ± 0.28 ^#^
Fecal						
TC	6.53 ± 1.01 **	10.74 ± 1.48	11.67 ± 0.83	15.12 ± 1.69 **	14.60 ± 2.04 **	12.04 ± 1.94 *
TG	6.40 ± 1.42 **	12.50 ± 0.96	13.63 ± 1.11	15.62 ± 1.80 *	15.32 ± 2.04 *	13.24 ± 2.72
TBA	1.83 ± 0.53 **	3.20 ± 0.91	2.90 ± 0.67	4.46 ± 1.25 *	4.40 ± 1.25*	4.58 ± 1.41 *

Data are expressed as means ± SD (*n* = 10 per group). (*^,#^) Significant difference at *p* < 0.05 vs HF group. Note: Compared with HF; * *p*, ^#^ *p* < 0.05, ** *p*, ^##^ *p* < 0.01, and *** *p*, ^###^ *p* < 0.001.

**Table 2:** Primer pairs were used for PCR.

Primers	Forward Primer (5′-3′)	Reverse Primer (5′-3′)
CYP7A1	ACCTGCCGGTACTAGACAGC	CAGGACATATTGT CGCGCCT
LDLR	GCCGACCTGACGAATTCCAG	ATCCGACCAGTCACGACAGT
LXRA	CTGCAACGGAGTTGTGGAAG	TCGCAGCTCAGCACATTGTA
SREBP2	GGAGACCATGGAGACCCTCAC	AGACAATGGGACCTGGCTGAA
HMGCR	CCTCC ATTGAGATCCGGAGGA	ACAAAGAGGCCATGCATACGG
PPARα	TCTGA ACATTGGCGTTCGCA+	TCCCTCAAGGGG ACA ACCAG
